# Anterior high-resolution OCT in the diagnosis and management of corneal squamous hyperplasia mimicking a malignancy: a case report

**DOI:** 10.1186/s12886-019-1237-4

**Published:** 2019-11-21

**Authors:** Yi-Syun Shen, Joseph L. Hu, Chao-Chien Hu

**Affiliations:** 1 Department of Ophthalmology, Shin Kong Wu Ho-Su Memorial Hospital, No. 95, Wen-Chang Road, Shih-Lin District, Taipei, 11120 Taiwan; 2 Hsin Ho Mei Eye Clinic, Songshan Branch, Taipei, Taiwan; 3 Department of Medicine, National Cheng Kung University College of Medicine, Tainan, Taiwan; 4 School of Medicine, Fu Jen Catholic University, Taipei, Taiwan; 5 School of Medicine, Taipei Medical University, Taipei, Taiwan

**Keywords:** Anterior high-resolution optical coherence tomography, Corneal squamous hyperplasia

## Abstract

**Background:**

Anterior high-resolution optical coherence tomography (HR-OCT) is a novel non-invasive in vivo imaging modality that can assist in the diagnosis and management of various ophthalmic pathologies. The implementation of diagnosing ocular surface lesions has been explored in previous studies, successfully revealing specific signs in some ocular lesions. This case report aims to exhibit a case of corneal squamous hyperplasia diagnosed via anterior HR-OCT, prior to surgical intervention.

**Case presentation:**

A 69 year-old male had blurred vision and foreign body sensation OD for several weeks. A rapidly-grown corneal mass was presented, showing an appearance of a grayish flesh-colored mass with elastic texture. Large vessels supplying the mass were also found. Anterior HR-OCT was performed, and the results suggested the lesion be benign hyperplasia. Superficial keratectomy was done, and the pathologic report showed mild-appearing epithelial squamous hyperplasia, which confirmed the analysis via anterior HR-OCT.

**Conclusion:**

In the categorization by Nanji, et al. of corneal surface diseases using anterior OCT, the comparative epithelial thickness (normal range: 47—68 μm); inferior border obscuration of epithelium (normal or benign inferior border: no shadowing); reflectivity of epithelial layer (normal: not hyper-reflective); abrupt transition (normal: no horizontally abrupt transition); and sub-epithelium analysis vary between benign and malignant lesions (normal: demarcated anterior to Bowman’s layer), and the differences are systemically sorted. We applied all these characteristics to our patient as guidance, and the measurement results indicated the lesion be a benign lesion, which is consistent with the tissue pathology. Anterior HR-OCT is overall a non-invasive and timely method capable of assisting the diagnosis of ocular surface disease, predicting the qualities of a lesion, and determining the follow-up treatment plan.

## Background

Anterior high-resolution optical coherence tomography (HR-OCT) is a novel non-invasive in vivo imaging modality that can assist in the diagnosis and management of various ophthalmic pathologies [1, 2]. The detection and diagnosis of ocular surface lesions, one of the many implementations of HR-OCT, has been explored in previous studies, successfully revealing optically-diagnostic features with an axial resolution at up to 3 μm [3]. The high resolution granted by HR-OCT not only renders the measurement and analysis of the thickness, reflectivity, and inferior border obscuration of epithelial layers capable, but also visualizes abrupt transitions of epithelial and sub-epithelial layers, allowing for the differentiation of malignancy from normality. This case report aims to exhibit a case with corneal squamous hyperplasia diagnosed with anterior HR-OCT prior to surgical intervention.

## Case presentation

**Fig. 1:**
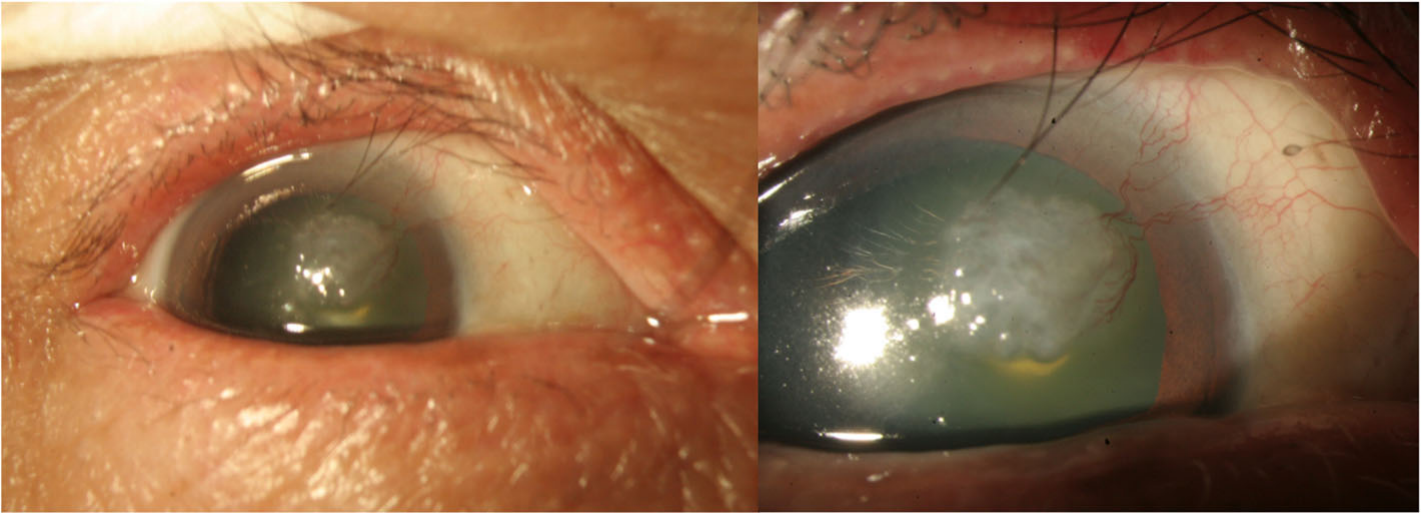
Gross picture of a corneal mass with fleshy color. Large limbal vessels supplying the lesion were noted. Right upper eyelid trichiasis over the medial side was also noted

A 69 year-old male with type 2 Diabetes Mellitus (DM) and hyperlipidemia presented to our out-patient department with complaints of blurry vision and foreign body sensation in his right eye over the past 2 to 3 months. The best-corrected visual acuity (BCVA) at presentation was OD: 20/40 and OS: 20/25, and bilateral upper eyelid trichiasis had also been noted for 2 years. In his right eye, an elevated mass over the naso-upper cornea was seen by direct visualization. Under slit lamp examination, the gross appearance of the lesion showed an elastic consistency with mild grayish and fleshy color, measuring approximately 6–7 mm in width. Large limbal vessels supplying the mass from the nasal side were also found (Fig. 1).

**Fig. 2:**
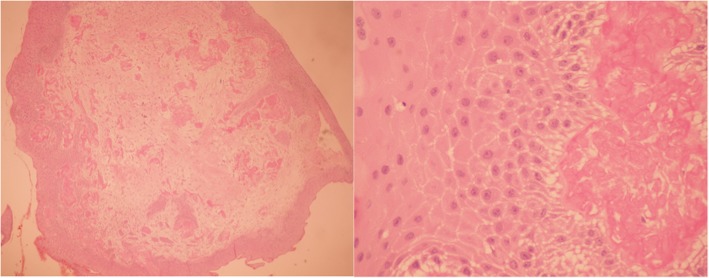
Pathologic sections revealed mild-looking squamous epithelia; the submucosa revealed fibrosis and focal neutrophilic cells infiltration. Squamous hyperplasia was noted while cellular atypia was absent. Left picture: low-power field. Right picture: high-power field

Anterior HR-OCT was performed on the patient using the AngioVue Avanti OCTA system (Optovue, Inc., Fremont, CA). The results showed that the lesion was demarcated anterior to Bowman’s layer without epithelial inferior border shadowing (Fig. 5). Epithelial thickness of the lesion ranged from 69 to 86 μm (Fig. 4). Mild hyper-reflectivity of the sub-epithelium was observed, while abrupt transition of the epithelium was absent (Figs. 5 and 6). According to the categorization of Nanji, et al. for corneal surface diseases, the information given by anterior HR-OCT suggested the lesion be benign hyperplasia. Superficial keratectomy followed by 20% alcohol soaking was performed to remove the lesion, and the specimen was sent for pathologic analysis. Pathologic reports showed mild-appearing epithelial squamous hyperplasia, submucosa fibrosis, foci of fibrinoid necrosis, and focal neutrophilic cells infiltration. Neither obvious cellular atypia nor evidence of malignancy was found (Fig. 2). This confirmed the analysis via anterior HR-OCT.

**Fig. 3:**
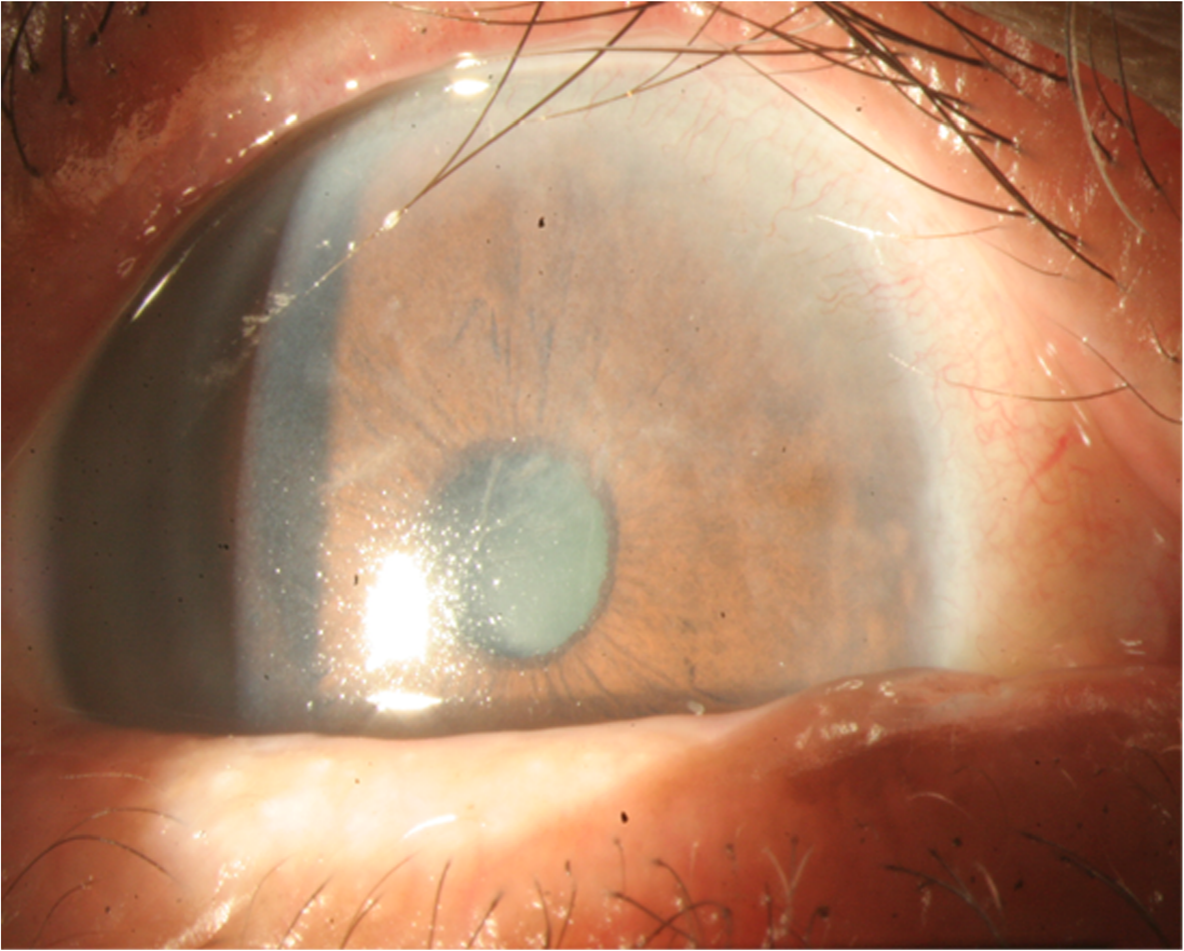
One week after keratectomy, the lesion mostly healed with only mild corneal edema remaining

Topical eyedrops of 0.25% chloramphenicol QID and 0.1% fluorometholone QID were prescribed. In the 1 week follow-up, the wound had mostly healed, leaving only a mildly irregular epithelial surface (Fig. 3).

## Discussion and conclusion

In this case, preoperative anterior HR-OCT, given its ability to visualize anterior-segment non-invasively, was shown useful in the process of delivering a more appropriate surgical treatment plan.

At presentation, the original appearance of the lesion was reminiscent of malignancy due to the gelatinous surface, irregular borders, rapid growth of the mass, and several supplying neo-vessels [4, 5]. The adoption of anterior HR-OCT was called for to further diagnose the patient.

**Fig. 4:**
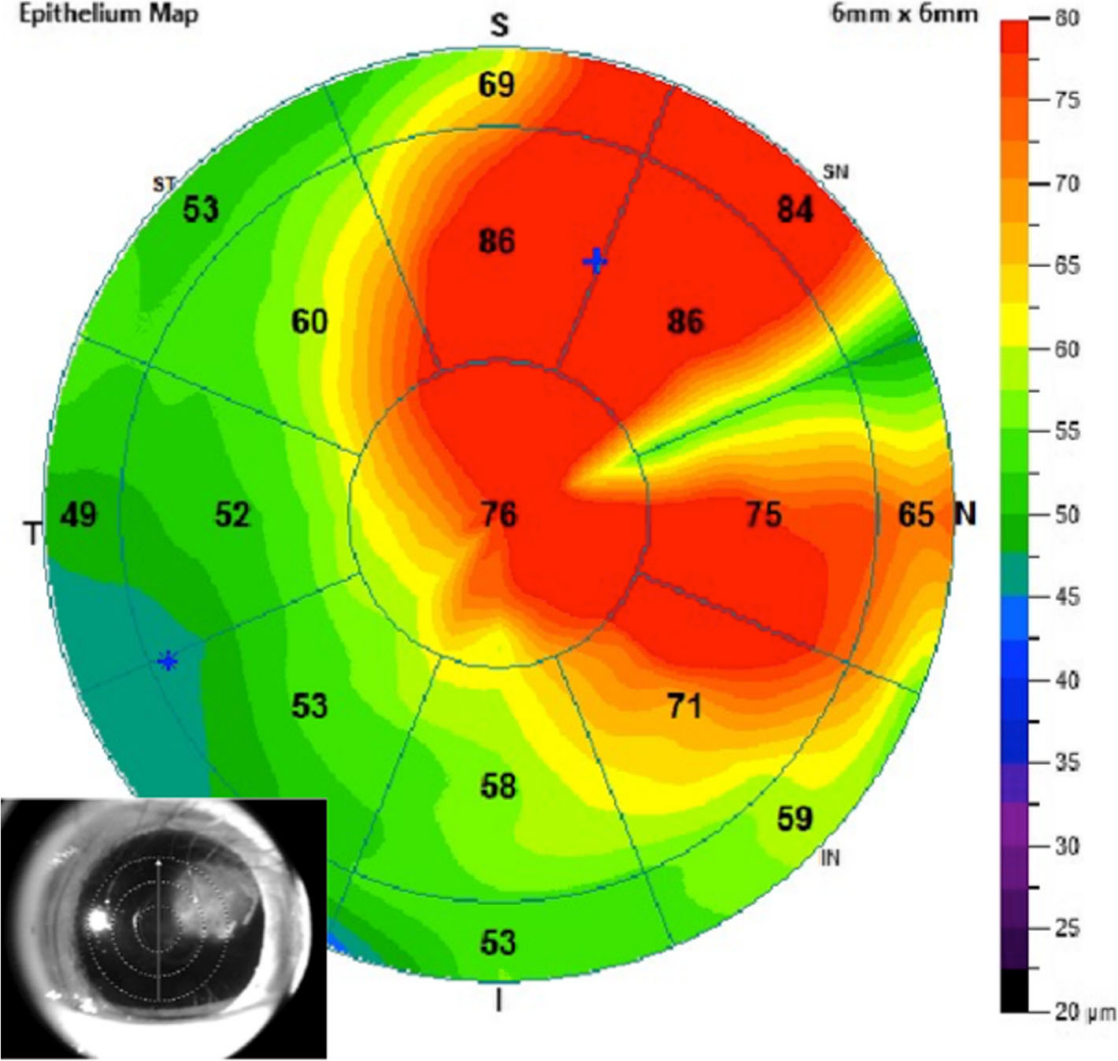
Epithelial map measured via anterior HR-OCT. Normal epithelial thickness are presented as green, ranging from 49 to 60 μm.Thickened epithelium ranging from 65 to 86 μm are presented as red. The red-colored region over the naso-upper portion indicates the affected area

**Fig. 5:**
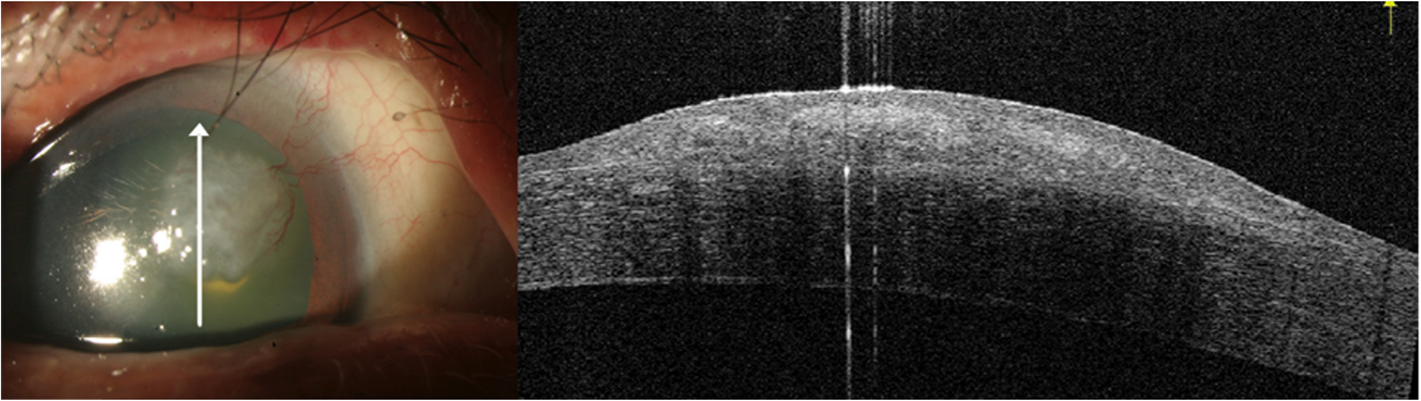
Anterior HR-OCT, vertical section. The picture reveals normal thickness and reflectivity of the epithelium and a dense hyper-reflective sub-epithelial lesion. There was no obviously abrupt transition from the lesion (elevated zone) to the normal area

**Fig. 6:**
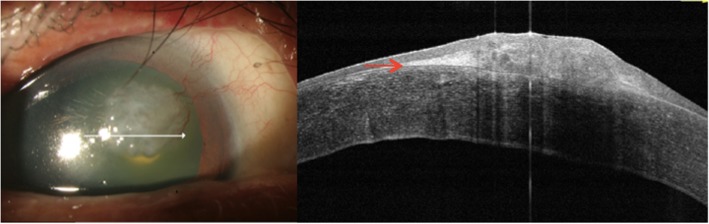
Anterior HR-OCT, horizontal section. The overlying epithelium is mostly homogenous. Despite the thickness being slightly increased, it was still within the range of a benign lesion (37 - 116 μm). The sub-epithelium tissue located anterior to Bowman’s layer (the bright demarcating line, red arrow), showed a dense, limited and hyper-reflective fibrillary sub-epithelial lesion

The results of the anterior HR-OCT revealed that the epithelial thickness of the lesion showed only a slight or mild increase, ranging from 69 to 86 μm (86 μm at the thickest) (Fig. 4). The lesion revealed no inferior obscuration by the thickened epithelium, and was confined anterior to Bowman’s layer on anterior HR-OCT. No obviously abrupt transition of the epithelium was noted between the normal and abnormal zone (Figs. 5 and 6). Mild hyper-reflectivity of the sub-epithelium was found (see Additional file 1: DataS1 and S2). Our results from HR-OCT analysis confirmed it to be a benign lesion, and it was consistent with the tissue pathology.

In Nanji, et al.’s analysis via anterior HR-OCT, a normal cornea epithelial layer would be approximately 54.5 μm in thickness (range 47 - 68 μm); in benign lesions such as pterygia, the epithelium would be normal to slightly thickened to around 69 μm (range 37 - 116 μm, under a safe cutoff value of 120 μm); and in malignancies such as ocular surface squamous neoplasia (OSSN) or corneal intraepithelial neoplasia (CIN), epithelial thickness increases to around 390 μm (range 124 - 1000 μm), and the inferior border of a malignant lesion may be partially obscured by shadowing, forming a darkened area under the thickened epithelium. The reflectivity of the epithelial layer of a malignant lesion is strongly hyper-reflective, whereas that of benign lesions such as pterygia or nodular degeneration is either normal or mildly hyper-reflective. In a malignant lesion, an abrupt transition from normal epithelium to a thickened and hyper-reflective epithelium is often observed, while the absence of an abrupt transition usually suggest a benign lesion.

Furthermore, in terms of sub-epithelium analysis, OSSN or CIN usually show no involvement, whereas benign lesions usually show dense, limited, and hyper-reflective fibrillary sub-epithelial lesions located anterior to Bowman’s layer [1, 2, 6]. This feature is compatible with histological findings such as pterygia, in which the fibroblastic lesions are demarcated by Bowman’s layer [7].

This is a real-world case study of benign hyperplasia evaluated by HR-OCT, and more studies are required to confirm the significance of this method. Cases on other types of malignant lesions, including OSSN or CIN, that are diagnosed with HR-OCT before pathologic proven can be applied for comparison to this case. In addition, to lower the possibility of misdiagnosis due to operational errors during the examination of HR-OCT, pathologic reports are still required for final confirmation.

Making an appropriate treatment plan in cases with atypical ocular lesion is difficult. However, the application of anterior HR-OCT shows efficacy in forming preliminary diagnosis, allowing practicing ophthalmologists to treat patients accurately and timely. Recently, the application of high-resolution OCT has been expanding swiftly and widely in ophthalmologic practices, and real-time imaging was even applied during ocular surgeries [8]. The adoption of such technique may not only improve the diagnostic rate, but also assist in localizing lesions and determining curettage depths. The adoption of OCT angiography in ocular surface lesion to distinguish the flow type of the feeding vessels is also promising, for it may provide more information to differentiate between benign and malignancy in the future [9].

Despite the suspicion of malignancy given by the clinical impressions in our case, we were able to differentiate the benign hyperplasia from malignancy accurately via anterior HR-OCT prior to operation. The final diagnosis of squamous hyperplasia was further affirmed by the pathology. This case showed the importance of HR-OCT for ophthalmologists by enabling decisions made at earlier stages. With the results at hand, practitioners can make decisions accordingly with different surgical plans and determine whether chemotherapy, such as mitomycin-C, be used during the peri-operative period [10]. Anterior HR-OCT is overall a non-invasive and timely method capable of assisting the diagnosis of ocular surface disease, predicting the qualities of a lesion, and determining the follow-up treatment plan.

## Supplementary information

**Additional file 1: Data S1.** Adjusted brightness of Fig. 5. The red circle is the transitional zone from the flat to the elevated surface. No obvious abrupt transition is noted. This picture also clearly demonstrates the dense, limited, and hyper-reflective sub-epithelial lesion. **Data S2.** Manual measurement of Fig. 6. Given the reference of 250 μm, the epithelial thickness, from right to left, was 94 μm, 90 μm, 101 μm, 105 μm, respectively. Though thicker than the epithelial map measured by anterior HR-OCT (range 65—86 μm in our case), this still falls within the benign range (37—116 μm).

## Data Availability

The datasets analyzed during the current study are available from the corresponding author on reasonable request.

## Abbreviations

BCVA: Best-corrected visual acuity
CIN: Corneal intraepithelial neoplasia
DM: Diabetes Mellitus
HR-OCT: High-resolution optical coherence tomography
OSSN: Ocular surface squamous neoplasia
