# Supplementary material for: Anterior high-resolution OCT in the diagnosis and management of corneal squamous hyperplasia mimicking a malignancy: a case report
Source: BMC Ophthalmol. 2019 Nov 21;19:235. doi: 10.1186/s12886-019-1237-4 (PMC6873769; doi:10.1186/s12886-019-1237-4)
Supplement: Supplementary file 1 — Additional file 1: Data S1. Adjusted brightness of Fig. 5. The red circle is the transitional zone from the flat to the elevated surface. No obvious abrupt transition is noted. This picture also clearly demonstrates the dense, limited, and hyper-reflective sub-epithelial lesion. Data S2. Manual measurement of Fig. 6. Given the reference of 250 μm, the epithelial thickness, from right to left, was 94 μm, 90 μm, 101 μm, 105 μm, respectively. Though thicker than the epithelial map measured by anterior HR-OCT (range 65—86 μm in our case), this still falls within the benign range (37—116 μm). [file 12886_2019_1237_MOESM1_ESM.docx]

**Additional file 1: Data S1**

**
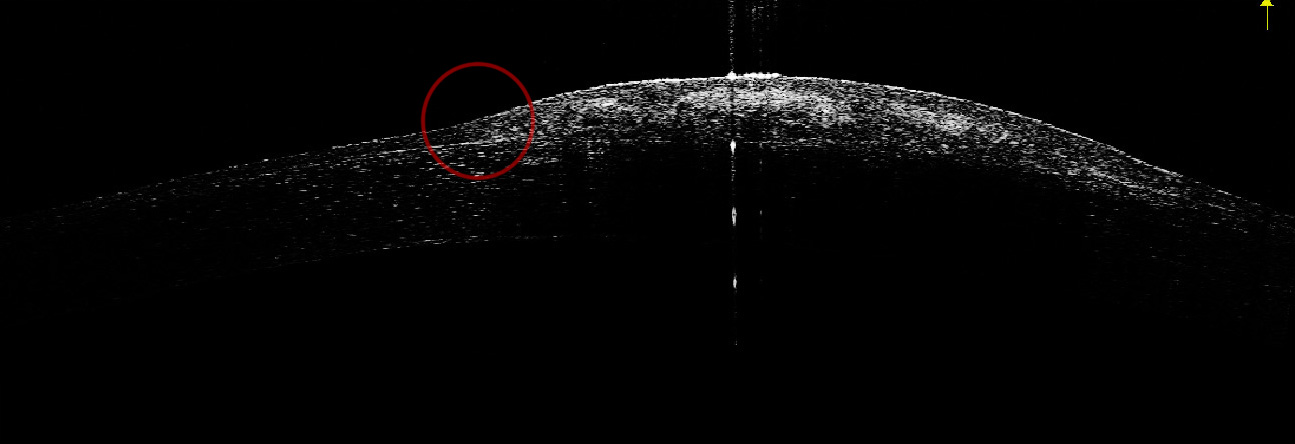
**

Adjusted brightness of figure 5. The red circle is the transitional zone from the flat to the elevated surface. No obvious abrupt transition is noted. This picture also clearly demonstrates the dense, limited, and hyper-reflective sub-epithelial lesion.

**Data S2**

**
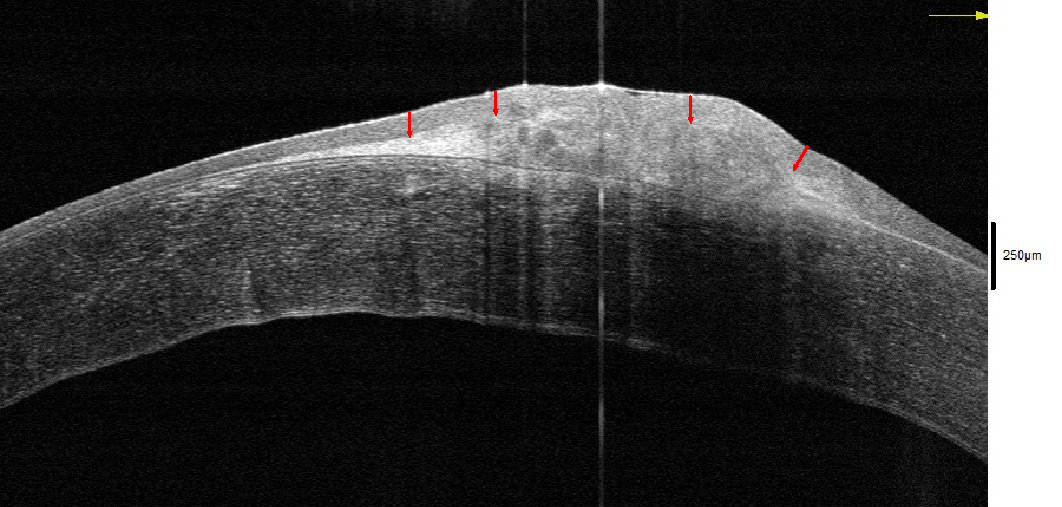
**

Manual measurement of figure 6. Given the reference of 250μm, the epithelial thickness, from right to left, was 94μm, 90μm, 101μm, 105μm, respectively. Though thicker than the epithelial map measured by anterior HR-OCT (range 65—86μm in our case), this still falls within the benign range (37—116μm).
